# Supplementary material for: Reciprocally inhibitory circuits operating with distinct mechanisms are differently robust to perturbation and modulation
Source: eLife. 2022 Feb 1;11:e74363. doi: 10.7554/eLife.74363 (PMC8884723; doi:10.7554/eLife.74363)
Supplement: Supplementary file 1. — (a) Mean ± SD of output characteristics of the circuits in escape and release at 10°C and 20°C. (b) Significance analysis of the cycle frequency, spike frequency, number of spikes per burst, slow wave amplitude, duty cycle and ERQ at 10°C and 20°C. (c-h) Significance analysis of the change in the output characteristics of the circuits in escape and release with different temperature-dependencies. [file elife-74363-supp1.docx]

**Supplementary File 1**

**Supplementary File 1a**

|  | **Escape** | | | | | | **Release** | | | | | |
| --- | --- | --- | --- | --- | --- | --- | --- | --- | --- | --- | --- | --- |
|  | **10^o^C** | | | **20^o^C** | | | **10^o^C** | | | **20^o^C** | | |
|  | $\boldsymbol{Q}_{\boldsymbol{10}}\boldsymbol{=1}$ | $\boldsymbol{Q}_{\boldsymbol{10}}\boldsymbol{=2}$**for** $\boldsymbol{g}_{\boldsymbol{H,}}\boldsymbol{g}_{\boldsymbol{Syn}}$ | $\boldsymbol{Q}_{\boldsymbol{10}}\boldsymbol{=2}$ | $\boldsymbol{Q}_{\boldsymbol{10}}\boldsymbol{=1}$ | $\boldsymbol{Q}_{\boldsymbol{10}}\boldsymbol{=2}$ **for** $\boldsymbol{g}_{\boldsymbol{H,}}\boldsymbol{g}_{\boldsymbol{Syn}}$ | $\boldsymbol{Q}_{\boldsymbol{10}}\boldsymbol{=2}$ | $\boldsymbol{Q}_{\boldsymbol{10}}\boldsymbol{=1}$ | $\boldsymbol{Q}_{\boldsymbol{10}}\boldsymbol{=2}$ **for** $\boldsymbol{g}_{\boldsymbol{H,}}\boldsymbol{g}_{\boldsymbol{Syn}}$ | $\boldsymbol{Q}_{\boldsymbol{10}}\boldsymbol{=2}$ | $\boldsymbol{Q}_{\boldsymbol{10}}\boldsymbol{=1}$ | $\boldsymbol{Q}_{\boldsymbol{10}}\boldsymbol{=2}$ **for** $\boldsymbol{g}_{\boldsymbol{H,}}\boldsymbol{g}_{\boldsymbol{Syn}}$ | $\boldsymbol{Q}_{\boldsymbol{10}}\boldsymbol{=2}$ |
| **Cycle freq, Hz** | $0.23\pm0.07$ | $0.20\pm0.09$ | $0.20\pm0.07$ | $0.23\pm0.08$ | $0.20\pm0.09$ | $0.46\pm0.19$ | $0.19\pm0.05$ | $0.23\pm0.09$ | $0.26\pm0.11$ | $0.28\pm0.09$ | $0.14\pm0.06$ | $0.30\pm0.11$ |
| **Spike freq, Hz** | $7.9\pm2.9$ | $6.6\pm2.1$ | $6.7\pm1.1$ | $10.1\pm4.8$ | $13.7\pm4.3$ | $19.4\pm2.0$ | $7.7\pm4.1$ | $5.9\pm2.8$ | $7.9\pm1.8$ | $9.1\pm4.6$ | $10.4\pm3.7$ | $15.7\pm3.1$ |
| **Amplitude, mV** | $22.7\pm6.3$ | $21.6\pm1.0$ | $21.8\pm2.4$ | $21.5\pm6.2$ | $27.5\pm4.4$ | $23.8\pm4.4$ | $27.9\pm4.8$ | $20.0\pm7.3$ | $19.6\pm5.3$ | $20.6\pm4.3$ | $32.7\pm10.3$ | $30.3\pm10.3$ |
| **# spikes/burst** | $9\pm5$ | $7\pm1$ | $7\pm2$ | $13\pm8$ | $18\pm5$ | $9\pm4$ | $18\pm13$ | $12\pm9$ | $14\pm6$ | $15\pm11$ | $36\pm23$ | $25\pm7$ |
| **Duty cycle, %** | $25.8\pm9.9$ | $20.1\pm5.2$ | $17.4\pm0.4$ | $26.7\pm10.2$ | $24.6\pm5.5$ | $17.0\pm1.6$ | $42.0\pm4.6$ | $36.2\pm10.6$ | $41.7\pm4.4$ | $40.0\pm5.3$ | $39.5\pm6.2$ | $44.8\pm2.9$ |
| **ERQ** | $-0.09\pm0.03$ | $-0.07\pm0.03$ | $-0.08\pm0.05$ | $-0.07\pm0.04$ | $-0.09\pm0.03$ | $-0.10\pm0.04$ | $0.13\pm0.06$ | $0.16\pm0.07$ | $0.14\pm0.07$ | $0.14\pm0.05$ | $0.15\pm0.08$ | $0.14\pm0.08$ |

**Supplementary File 1b**

| **Paired-samples Wilcoxon signed rank test for cycle frequency at 10^o^C vs 20^o^C**   \| **Mechanism** \| $\boldsymbol{Q}_{\boldsymbol{10}}$ \| **N** \| **p-value** \| \| --- \| --- \| --- \| --- \| \| **Escape** \| $1$ \| 11 \| .100 \| \| $2 for g_{H,}g_{Syn}$ \| 4 \| .144 \| \| $2$ \| 3 \| .109 \| \| **Release** \| $1$ \| 6 \| **.028** \| \| $2 for g_{H,}g_{Syn}$ \| 5 \| **.043** \| \| $2$ \| 4 \| .144 \| | **Paired-samples Wilcoxon signed rank test for spike frequency at 10^o^C vs 20^o^C**   \| **Mechanism** \| $\boldsymbol{Q}_{\boldsymbol{10}}$ \| **N** \| **p-value** \| \| --- \| --- \| --- \| --- \| \| **Escape** \| $1$ \| 21 \| **<.001** \| \| $2 for g_{H,}g_{Syn}$ \| 8 \| **.012** \| \| $2$ \| 6 \| **.028** \| \| **Release** \| $1$ \| 12 \| **.003** \| \| $2 for g_{H,}g_{Syn}$ \| 10 \| **.005** \| \| $2$ \| 8 \| **.012** \| |
| --- | --- | --- | --- | --- | --- | --- | --- | --- | --- | --- | --- | --- | --- | --- | --- | --- | --- | --- | --- | --- | --- | --- | --- | --- | --- | --- | --- | --- | --- | --- | --- | --- | --- | --- | --- | --- | --- | --- | --- | --- | --- | --- | --- | --- | --- | --- | --- | --- | --- |
| **Paired-samples Wilcoxon signed rank test for # spikes/burst at 10^o^C vs 20^o^C**   \| **Mechanism** \| $\boldsymbol{Q}_{\boldsymbol{10}}$ \| **N** \| **p-value** \| \| --- \| --- \| --- \| --- \| \| **Escape** \| $1$ \| 21 \| **<.001** \| \| $2 for g_{H,}g_{Syn}$ \| 8 \| **0.018** \| \| $2$ \| 6 \| .246 \| \| **Release** \| $1$ \| 12 \| **.041** \| \| $2 for g_{H,}g_{Syn}$ \| 10 \| **.005** \| \| $2$ \| 8 \| **.018** \| | **Paired-samples Wilcoxon signed rank test for amplitude at 10^o^C vs 20^o^C**   \| **Mechanism** \| $\boldsymbol{Q}_{\boldsymbol{10}}$ \| **N** \| **p-value** \| \| --- \| --- \| --- \| --- \| \| **Escape** \| $1$ \| 21 \| **.011** \| \| $2 for g_{H,}g_{Syn}$ \| 8 \| **.012** \| \| $2$ \| 6 \| .075 \| \| **Release** \| $1$ \| 12 \| **.002** \| \| $2 for g_{H,}g_{Syn}$ \| 10 \| **.005** \| \| $2$ \| 8 \| **.012** \| |
| **Paired-samples Wilcoxon signed rank test for duty cycle at 10^o^C vs 20^o^C**   \| **Mechanism** \| $\boldsymbol{Q}_{\boldsymbol{10}}$ \| **N** \| **p-value** \| \| --- \| --- \| --- \| --- \| \| **Escape** \| $1$ \| 21 \| .590 \| \| $2 for g_{H,}g_{Syn}$ \| 8 \| .123 \| \| $2$ \| 6 \| .753 \| \| **Release** \| $1$ \| 12 \| .388 \| \| $2 for g_{H,}g_{Syn}$ \| 10 \| .646 \| \| $2$ \| 8 \| **.036** \| | **Paired-samples Wilcoxon signed rank test for ERQ at 10^o^C vs 20^o^C**   \| **Mechanism** \| $\boldsymbol{Q}_{\boldsymbol{10}}$ \| **N** \| **p-value** \| \| --- \| --- \| --- \| --- \| \| **Escape** \| $1$ \| 21 \| **.009** \| \| $2 for g_{H,}g_{Syn}$ \| 8 \| **.017** \| \| $2$ \| 6 \| .173 \| \| **Release** \| $1$ \| 12 \| .136 \| \| $2 for g_{H,}g_{Syn}$ \| 10 \| .286 \| \| $2$ \| 8 \| .575 \| |

**Supplementary File 1c** **Supplementary File 1d**   **Supplementary File 1e**

| \| **Measure: change in cycle frequency from 10^o^C to 20^o^C; Test: One-way ANOVA; F-statistic: F(5,59)=21.790, p<0.001; Post-hoc: Tuckey** \| \| \| \| \| \| \| \| \| --- \| --- \| --- \| --- \| --- \| --- \| --- \| --- \| \|  \|  \| **Escape** \| \| \| **Release** \| \| \| \|  \| $\boldsymbol{Q}_{\boldsymbol{10}}$ \| $\boldsymbol{1}\boldsymbol{all}$ \| $\boldsymbol{2 for}$ $\boldsymbol{g}_{\boldsymbol{s,}}\boldsymbol{g}_{\boldsymbol{Syn}}$ \| $\boldsymbol{2 all}$ \| $\boldsymbol{1}\boldsymbol{all}$ \| $\boldsymbol{2 for}$ $\boldsymbol{g}_{\boldsymbol{s,}}\boldsymbol{g}_{\boldsymbol{Syn}}$ \| $\boldsymbol{2 all}$ \| \| **Escape** \| $\boldsymbol{1}\boldsymbol{all}$ \|  \| .999 \| **<.001** \| **.014** \| **.053** \| .540 \| \| $\boldsymbol{2}$ **for** $\boldsymbol{g}_{\boldsymbol{H,}}\boldsymbol{g}_{\boldsymbol{Syn}}$ \|  \|  \| **<.001** \| .147 \| .101 \| .864 \| \| $\boldsymbol{2 all}$ \|  \|  \|  \| **<.001** \| **<.001** \| **<.001** \| \| **Release** \| $\boldsymbol{1}\boldsymbol{all}$ \|  \|  \|  \|  \| **<.001** \| .781 \| \| $\boldsymbol{2}$ **for** $\boldsymbol{g}_{\boldsymbol{H,}}\boldsymbol{g}_{\boldsymbol{Syn}}$ \|  \|  \|  \|  \|  \| **.006** \| \| $\boldsymbol{2 all}$ \|  \|  \|  \|  \|  \|  \|   **Supplementary File 1f** | \| **Measure: change in spike frequency from 10^o^C to 20^o^C; Test: One-way ANOVA; F-statistic: F(5,59)=9.897, p<0.001; Post-hoc: Tuckey** \| \| \| \| \| \| \| \| \| --- \| --- \| --- \| --- \| --- \| --- \| --- \| --- \| \|  \|  \| **Escape** \| \| \| **Release** \| \| \| \|  \| $\boldsymbol{Q}_{\boldsymbol{10}}$ \| $\boldsymbol{1}\boldsymbol{all}$ \| $\boldsymbol{2 for}$ $\boldsymbol{g}_{\boldsymbol{s,}}\boldsymbol{g}_{\boldsymbol{Syn}}$ \| $\boldsymbol{2 all}$ \| $\boldsymbol{1}\boldsymbol{all}$ \| $\boldsymbol{2 for}$ $\boldsymbol{g}_{\boldsymbol{s,}}\boldsymbol{g}_{\boldsymbol{Syn}}$ \| $\boldsymbol{2 all}$ \| \| **Escape** \| $\boldsymbol{1}\boldsymbol{all}$ \|  \| **.023** \| **<.001** \| .981 \| .580 \| **.006** \| \| $\boldsymbol{2}$ **for** $\boldsymbol{g}_{\boldsymbol{H,}}\boldsymbol{g}_{\boldsymbol{Syn}}$ \|  \|  \| .276 \| **.011** \| .660 \| .999 \| \| $\boldsymbol{2 all}$ \|  \|  \|  \| **<.001** \| **.007** \| .483 \| \| **Release** \| $\boldsymbol{1}\boldsymbol{all}$ \|  \|  \|  \|  \| .324 \| **.003** \| \| $\boldsymbol{2}$ **for** $\boldsymbol{g}_{\boldsymbol{H,}}\boldsymbol{g}_{\boldsymbol{Syn}}$ \|  \|  \|  \|  \|  \| .397 \| \| $\boldsymbol{2 all}$ \|  \|  \|  \|  \|  \|  \|   **Supplementary File 1g** | \| **Measure: change in # spikes/burst from 10^o^C to 20^o^C; Test: One-way ANOVA; F-statistic: F(5,59)=13.949, p<0.001; Post-hoc: Tuckey** \| \| \| \| \| \| \| \| \| --- \| --- \| --- \| --- \| --- \| --- \| --- \| --- \| \|  \|  \| **Escape** \| \| \| **Release** \| \| \| \|  \| $\boldsymbol{Q}_{\boldsymbol{10}}$ \| $\boldsymbol{1}\boldsymbol{all}$ \| $\boldsymbol{2 for}$ $\boldsymbol{g}_{\boldsymbol{s,}}\boldsymbol{g}_{\boldsymbol{Syn}}$ \| $\boldsymbol{2 all}$ \| $\boldsymbol{1}\boldsymbol{all}$ \| $\boldsymbol{2 for}$ $\boldsymbol{g}_{\boldsymbol{s,}}\boldsymbol{g}_{\boldsymbol{Syn}}$ \| $\boldsymbol{2 all}$ \| \| **Escape** \| $\boldsymbol{1}\boldsymbol{all}$ \|  \| .229 \| .999 \| .286 \| **<.001** \| .279 \| \| $\boldsymbol{2}$ **for** $\boldsymbol{g}_{\boldsymbol{H,}}\boldsymbol{g}_{\boldsymbol{Syn}}$ \|  \|  \| .354 \| **.005** \| **.020** \| 1.00 \| \| $\boldsymbol{2 all}$ \|  \|  \|  \| .818 \| **<.001** \| .391 \| \| **Release** \| $\boldsymbol{1}\boldsymbol{all}$ \|  \|  \|  \|  \| **<.001** \| **.007** \| \| $\boldsymbol{2}$ **for** $\boldsymbol{g}_{\boldsymbol{H,}}\boldsymbol{g}_{\boldsymbol{Syn}}$ \|  \|  \|  \|  \|  \| **.015** \| \| $\boldsymbol{2 all}$ \|  \|  \|  \|  \|  \|  \|   **Supplementary File 1h** |
| --- | --- | --- | --- | --- | --- | --- | --- | --- | --- | --- | --- | --- | --- | --- | --- | --- | --- | --- | --- | --- | --- | --- | --- | --- | --- | --- | --- | --- | --- | --- | --- | --- | --- | --- | --- | --- | --- | --- | --- | --- | --- | --- | --- | --- | --- | --- | --- | --- | --- | --- | --- | --- | --- | --- | --- | --- | --- | --- | --- | --- | --- | --- | --- | --- | --- | --- | --- | --- | --- | --- | --- | --- | --- | --- | --- | --- | --- | --- | --- | --- | --- | --- | --- | --- | --- | --- | --- | --- | --- | --- | --- | --- | --- | --- | --- | --- | --- | --- | --- | --- | --- | --- | --- | --- | --- | --- | --- | --- | --- | --- | --- | --- | --- | --- | --- | --- | --- | --- | --- | --- | --- | --- | --- | --- | --- | --- | --- | --- | --- | --- | --- | --- | --- | --- | --- | --- | --- | --- | --- | --- | --- | --- | --- | --- | --- | --- | --- | --- | --- | --- | --- | --- | --- | --- | --- | --- | --- | --- | --- | --- | --- | --- | --- | --- | --- | --- | --- | --- | --- | --- | --- | --- | --- | --- | --- | --- | --- | --- | --- | --- | --- | --- | --- | --- | --- | --- | --- | --- | --- | --- | --- | --- | --- | --- | --- | --- | --- | --- | --- | --- | --- | --- | --- | --- | --- | --- |
| \| **Measure: change in amplitude from 10^o^C to 20^o^C; Test: One-way ANOVA; F-statistic: F(5,59)=50.437, p<0.001; Post-hoc: Tuckey** \| \| \| \| \| \| \| \| \| --- \| --- \| --- \| --- \| --- \| --- \| --- \| --- \| \|  \|  \| **Escape** \| \| \| **Release** \| \| \| \|  \| $\boldsymbol{Q}_{\boldsymbol{10}}$ \| $\boldsymbol{1}\boldsymbol{all}$ \| $\boldsymbol{2 for}$ $\boldsymbol{g}_{\boldsymbol{s,}}\boldsymbol{g}_{\boldsymbol{Syn}}$ \| $\boldsymbol{2 all}$ \| $\boldsymbol{1}\boldsymbol{all}$ \| $\boldsymbol{2 for}$ $\boldsymbol{g}_{\boldsymbol{s,}}\boldsymbol{g}_{\boldsymbol{Syn}}$ \| $\boldsymbol{2 all}$ \| \| **Escape** \| $\boldsymbol{1}\boldsymbol{all}$ \|  \| **<.001** \| .406 \| **<.001** \| **<.001** \| **<.001** \| \| $\boldsymbol{2}$ **for** $\boldsymbol{g}_{\boldsymbol{H,}}\boldsymbol{g}_{\boldsymbol{Syn}}$ \|  \|  \| .314 \| **<.001** \| **.002** \| .082 \| \| $\boldsymbol{2 all}$ \|  \|  \|  \| **<.001** \| **<.001** \| **<.001** \| \| **Release** \| $\boldsymbol{1}\boldsymbol{all}$ \|  \|  \|  \|  \| **<.001** \| **<.001** \| \| $\boldsymbol{2}$ **for** $\boldsymbol{g}_{\boldsymbol{H,}}\boldsymbol{g}_{\boldsymbol{Syn}}$ \|  \|  \|  \|  \|  \| .853 \| \| $\boldsymbol{2 all}$ \|  \|  \|  \|  \|  \|  \| | \| **Measure: change in duty cycle from 10^o^C to 20^o^C; Test: One-way ANOVA; F-statistic: F(5,59)= 0.964, p=0.447; Post-hoc: Tuckey** \| \| \| \| \| \| \| \| \| --- \| --- \| --- \| --- \| --- \| --- \| --- \| --- \| \|  \|  \| **Escape** \| \| \| **Release** \| \| \| \|  \| $\boldsymbol{Q}_{\boldsymbol{10}}$ \| $\boldsymbol{1}\boldsymbol{all}$ \| $\boldsymbol{2 for}$ $\boldsymbol{g}_{\boldsymbol{s,}}\boldsymbol{g}_{\boldsymbol{Syn}}$ \| $\boldsymbol{2 all}$ \| $\boldsymbol{1}\boldsymbol{all}$ \| $\boldsymbol{2 for}$ $\boldsymbol{g}_{\boldsymbol{s,}}\boldsymbol{g}_{\boldsymbol{Syn}}$ \| $\boldsymbol{2 all}$ \| \| **Escape** \| $\boldsymbol{1}\boldsymbol{all}$ \|  \| .821 \| 1.00 \| .961 \| .939 \| .964 \| \| $\boldsymbol{2}$ **for** $\boldsymbol{g}_{\boldsymbol{H,}}\boldsymbol{g}_{\boldsymbol{Syn}}$ \|  \|  \| .865 \| .489 \| .999 \| .999 \| \| $\boldsymbol{2 all}$ \|  \|  \|  \| .999 \| .948 \| .964 \| \| **Release** \| $\boldsymbol{1}\boldsymbol{all}$ \|  \|  \|  \|  \| .647 \| .729 \| \| $\boldsymbol{2}$ **for** $\boldsymbol{g}_{\boldsymbol{H,}}\boldsymbol{g}_{\boldsymbol{Syn}}$ \|  \|  \|  \|  \|  \| .000 \| \| $\boldsymbol{2 all}$ \|  \|  \|  \|  \|  \|  \| | \| **Measure: change in ERQ from 10^o^C to 20^o^C; Test: One-way ANOVA; F-statistic: F(5,59)=4.076, p=0.003; Post-hoc: Tuckey** \| \| \| \| \| \| \| \| \| --- \| --- \| --- \| --- \| --- \| --- \| --- \| --- \| \|  \|  \| **Escape** \| \| \| **Release** \| \| \| \|  \| $\boldsymbol{Q}_{\boldsymbol{10}}$ \| $\boldsymbol{1}\boldsymbol{all}$ \| $\boldsymbol{2 for}$ $\boldsymbol{g}_{\boldsymbol{s,}}\boldsymbol{g}_{\boldsymbol{Syn}}$ \| $\boldsymbol{2 all}$ \| $\boldsymbol{1}\boldsymbol{all}$ \| $\boldsymbol{2 for}$ $\boldsymbol{g}_{\boldsymbol{s,}}\boldsymbol{g}_{\boldsymbol{Syn}}$ \| $\boldsymbol{2 all}$ \| \| **Escape** \| $\boldsymbol{1}\boldsymbol{all}$ \|  \| **.019** \| **.033** \| .970 \| 1.00 \| .549 \| \| $\boldsymbol{2}$ **for** $\boldsymbol{g}_{\boldsymbol{H,}}\boldsymbol{g}_{\boldsymbol{Syn}}$ \|  \|  \| 1.00 \| .177 \| **.040** \| .757 \| \| $\boldsymbol{2 all}$ \|  \|  \|  \| .208 \| **.054** \| .747 \| \| **Release** \| $\boldsymbol{1}\boldsymbol{all}$ \|  \|  \|  \|  \| .963 \| .942 \| \| $\boldsymbol{2}$ **for** $\boldsymbol{g}_{\boldsymbol{H,}}\boldsymbol{g}_{\boldsymbol{Syn}}$ \|  \|  \|  \|  \|  \| .592 \| \| $\boldsymbol{2 all}$ \|  \|  \|  \|  \|  \|  \| |
