# Supplementary material for: Reciprocally inhibitory circuits operating with distinct mechanisms are differently robust to perturbation and modulation
Source: eLife. 2022 Feb 1;11:e74363. doi: 10.7554/eLife.74363 (PMC8884723; doi:10.7554/eLife.74363)
Supplement: Transparent reporting form [file elife-74363-transrepform1.docx]

***eLife’s* transparent reporting form**

We encourage authors to provide detailed information *within their submission* to facilitate the interpretation and replication of experiments. Authors can upload supporting documentation to indicate the use of appropriate reporting guidelines for health-related research (see [EQUATOR Network](http://www.equator-network.org/%20)), life science research (see the [BioSharing Information Resource](https://biosharing.org/)), or the [ARRIVE guidelines](http://www.plosbiology.org/article/info:doi/10.1371/journal.pbio.1000412) for reporting work involving animal research. Where applicable, authors should refer to any relevant reporting standards documents in this form.

If you have any questions, please consult our Journal Policies and/or contact us: [editorial@elifesciences.org](mailto:editorial@elifesciences.org).

**Sample-size estimation**

- You should state whether an appropriate sample size was computed when the study was being designed
- You should state the statistical method of sample size computation and any required assumptions
- If no explicit power analysis was used, you should describe how you decided what sample (replicate) size (number) to use

Please outline where this information can be found within the submission (e.g., sections or figure legends), or explain why this information doesn’t apply to your submission:

No explicit power analysis to predetermine the sample size was performed as within group variability for each condition was relatively low, due to the controlled nature of the dynamic clamp experiments.

For the part of the study, the experimental design and the sample size estimation were based on the previously published dynamic-clamp half-center oscillator studies (Sharp et al., 1996, Grashow et al., 2008).

**Replicates**

- You should report how often each experiment was performed
- You should include a definition of biological versus technical replication
- The data obtained should be provided and sufficient information should be provided to indicate the number of independent biological and/or technical replicates
- If you encountered any outliers, you should describe how these were handled
- Criteria for exclusion/inclusion of data should be clearly stated
- High-throughput sequence data should be uploaded before submission, with a private link for reviewers provided (these are available from both GEO and ArrayExpress)

Please outline where this information can be found within the submission (e.g., sections or figure legends), or explain why this information doesn’t apply to your submission:

The number of times each type of experiment was performed is reported in the “Materials and Methods” section and in the figure legends.

We did not perform any outlier analysis, nor did we eliminate any data points based on this criterion.

All the experiments resulting in creation of stable half-center oscillators were included in the analysis. The procedure of building half-center oscillators is provided in the “Materials and Methods” section.

**Statistical reporting**

- Statistical analysis methods should be described and justified
- Raw data should be presented in figures whenever informative to do so (typically when N per group is less than 10)
- For each experiment, you should identify the statistical tests used, exact values of N, definitions of center, methods of multiple test correction, and dispersion and precision measures (e.g., mean, median, SD, SEM, confidence intervals; and, for the major substantive results, a measure of effect size (e.g., Pearson's r, Cohen's d)
- Report exact p-values wherever possible alongside the summary statistics and 95% confidence intervals. These should be reported for all key questions and not only when the p-value is less than 0.05.

Please outline where this information can be found within the submission (e.g., sections or figure legends), or explain why this information doesn’t apply to your submission:

All the details of statistical tests and significance analysis are described in the “Materials and Methods” (Statistics) section. The exact p-values (when appropriate), summary statistics (Means $\pm$ SD) and exact values of N are reported in the main text, figure legends and Supplementary tables S1-8.

(For large datasets, or papers with a very large number of statistical tests, you may upload a single table file with tests, Ns, etc., with reference to sections in the manuscript.)

**Group allocation**

- Indicate how samples were allocated into experimental groups (in the case of clinical studies, please specify allocation to treatment method); if randomization was used, please also state if restricted randomization was applied
- Indicate if masking was used during group allocation, data collection and/or data analysis

Please outline where this information can be found within the submission (e.g., sections or figure legends), or explain why this information doesn’t apply to your submission:

Half-center oscillators were allocated to release/escape or mixed mode oscillation mechanism group based on the position of the synaptic threshold within the slow wave, described in detail in Figure 2. The same half-center oscillator circuits were tested with various experimental conditions, for example, at different temperatures.

**Additional data files (“source data”)**

- We encourage you to upload relevant additional data files, such as numerical data that are represented as a graph in a figure, or as a summary table
- Where provided, these should be in the most useful format, and they can be uploaded as “Source data” files linked to a main figure or table
- Include model definition files including the full list of parameters used
- Include code used for data analysis (e.g., R, MatLab)
- Avoid stating that data files are “available upon request”

Please indicate the figures or tables for which source data files have been provided:

Custom RTXI module and analysis scripts are freely available on GitHub

<https://github.com/eomorozova/half_center_oscillator_rtxi_module>

<https://github.com/eomorozova/hco-analysis>
